# Characterizing Indicators of Engagement in HIV-Associated Healthcare and Clinical Outcomes among People with HIV and Mpox in Washington, DC: A Nested Case-Control Study of the DC Cohort

**DOI:** 10.3390/pathogens13020117

**Published:** 2024-01-27

**Authors:** Lauren F. O’Connor, Morgan Byrne, Anuja Baskaran, Elisabeth W. Andersen, Michael A. Horberg, Debra A. Benator, Jose Lucar, Rachel V. Denyer, Rachel Lee, Amanda D. Castel, Anne K. Monroe

**Affiliations:** 1 Milken Institute School of Public Health, The George Washington University, Washington, DC 20052, USA; 2 Kaiser Permanente Mid-Atlantic Permanente Medical Group, Rockville, MD 20852, USA; 3 District of Columbia Veterans Affairs Medical Center, Washington, DC 20422, USA; 4 School of Medicine & Health Sciences, The George Washington University, Washington, DC 20037, USA

**Keywords:** mpox, HIV, sexually transmitted infections, engagement in care, nested case-control study, HIV cohort

## Abstract

The high proportion of people with HIV (PWH) in the 2022–2023 mpox outbreak has raised questions surrounding the association between HIV and mpox. The objectives of this study were to evaluate the association between engagement in HIV-associated healthcare and mpox diagnosis, as well as to characterize cases of mpox among PWH. The DC Cohort is a longitudinal cohort of PWH in Washington, DC. We conducted a 5:1 (controls:cases) nested case-cohort study on male participants, matching age and care site. Cases were participants with an identified mpox diagnosis. Conditional logistic regression was used to assess the impact of indicators of engagement in HIV-associated healthcare on mpox diagnosis. We identified 70 cases of mpox in DC Cohort participants randomly matched to 323 controls, for a total of 393 participants included in the analysis. Study participants were primarily non-Hispanic Black (72.3%) with a median age of 41 (IQR: 36, 50). There was no association between engagement in care and mpox diagnosis; however, low CD4 was associated with increased odds of mpox diagnosis (aOR: 4.60 (95% CI: 1.23, 17.11)). Among a cohort of PWH, engagement in care was not associated with mpox diagnosis, suggesting that the overrepresentation of PWH among mpox cases is not due to surveillance bias.

## 1. Introduction

The world saw the first global outbreak of mpox (formerly known as “monkeypox”) in May 2022. As of May 2023, 30,422 cases have been reported in the United States (US), with 87,543 cases worldwide [1]. In Washington, DC, there have been 529 confirmed cases of mpox, most of which occurred in 2022 [2]. Most of these cases have occurred through sexual transmission, primarily among men who have sex with men (MSM) [3]. Among those with mpox with a known HIV status, 41% have been people with HIV (PWH) [2]. Previous studies have found evidence that low CD4 counts and high HIV RNA (viral load) among PWH are associated with more severe mpox outcomes [4,5,6]. These findings suggest mpox may act as an opportunistic infection among PWH, although this is not definitive [7,8]. The overrepresentation of PWH among mpox cases and the risk for severe mpox outcomes have raised questions over the relationship between HIV and mpox.

Previously, it has been suggested that the overrepresentation of PWH among mpox cases could be due to a relationship with sexually transmitted infections (STIs) or through increased encounters with the healthcare system leading to increased testing [9]. Although mpox is not currently classified as an STI, transmission of the virus in the 2022 outbreak has primarily occurred through sexual networks [3,10,11,12]. Common STIs such as gonorrhea, syphilis, and chlamydia have been prevalent among people with mpox, and PWH are known to be overrepresented among STI cases [6,13,14]. Therefore, high-risk sexual behaviors could be contributing to the high prevalence of mpox among PWH.

PWH may also be more likely to be diagnosed with mpox due to increased encounters with the healthcare system [9]. Most rashes appear in hard-to-see locations, such as the genital and anorectal regions [15,16]. Therefore, patients may not seek immediate care unless they are already interacting with the healthcare system. This trend in increased screenings due to healthcare encounters has been found previously. For example, a study by Kutner et al. among MSM and transgender women who have sex with men at high risk for STI acquisition found that a “routine doctor’s visit” was the most common reason for STI testing [17]. It is possible that PWH who already have a relationship with their healthcare provider may be more likely to be screened or seek care for mpox symptoms. However, there is currently not enough evidence to determine whether high utilization versus low utilization of health care, specifically HIV- or STI-associated care among PWH, is responsible for the increased case detection of mpox among high utilizers. 

To address these gaps in the literature and determine whether surveillance bias could be leading to higher rates of mpox diagnoses, we used data from the DC Cohort, a longitudinal cohort of PWH in Washington, DC, to evaluate the potential association between HIV-associated healthcare and receiving an mpox diagnosis. Given the incidence of mpox in Washington, DC, as well as the high prevalence of PWH, disparities in access to care, and available data on interactions with the healthcare system, the DC Cohort provides a unique landscape to evaluate these objectives [13]. The primary objective of this study was to determine whether recent indicators of engagement in HIV-associated healthcare are associated with having an mpox diagnosis among PWH receiving care in Washington, DC. Our secondary objective was to characterize the demographics, HIV clinical outcomes, and characteristics of PWH diagnosed with mpox in the DC Cohort.

## 2. Materials and Methods

### 2.1. Data Source

The data source for this analysis is the DC Cohort, a longitudinal cohort study of PWH at 14 clinical sites in Washington, DC. Enrollment for the DC Cohort began in January 2011 and includes limited manual data abstraction at baseline, automated extraction of electronic health record (EHR) data, and linkage with HIV surveillance data from the DC Department of Health. Details of the DC Cohort methodology have been described previously [18,19]. Eleven sites had ongoing EHR data extraction and were included at the time of the analysis. Participants were eligible to be included in the study if they were DC Cohort participants who identified as male and had evidence of HIV care between 2015–2023, which includes either an HIV-associated lab such as HIV RNA or CD4 count or an HIV-associated care visit. This 2015–2023 period was chosen to ensure that only actively enrolled participants were included in the analysis. Our analysis was restricted to males due to the limited number of mpox cases among non-male participants. Participants were excluded if they withdrew from the DC Cohort study, were deceased, or were transferred to another care site before the index date (defined below). The DC Cohort received approval from the George Washington University Institutional Review Board (IRB) (#071029). External participating sites with their own IRBs also obtained additional approval. Informed consent was provided by all participants during a routine clinical visit or while receiving care from a participating site where consent for data use for research purposes is part of clinical care and explained annually in the explanation of benefits. 

### 2.2. Case and Control Participants

A nested case-control study was conducted among PWH participating in the DC Cohort. Case participants were men who had a diagnosis of mpox during 2022, identified either by HIV care providers or from ICD10 codes extracted from the EHR. ICD10 code descriptions with terms referring to both mpox/monkeypox and general orthopoxvirus infections were considered positive for an mpox diagnosis. Each case was matched based on age (+/− 5 years) and site of care to five control participants using incidence density sampling based on calendar time. The index date for cases was the date of mpox diagnosis; for controls, it was the date of their matched case’s mpox diagnosis. Per incidence density sampling methodology, participants with mpox were eligible to be chosen as controls if their mpox diagnosis did not precede that of the index case. 

### 2.3. Primary Exposures of Interest: Engagement in HIV-Associated Care and STI Screening

Our primary objective was to determine whether indicators of engagement in care are associated with mpox diagnosis. Therefore, the primary exposures of interest were engagement in HIV-associated care and STI screening in the year before mpox diagnosis. The 12-month assessment period ended 10 days before the index date. This 10-day window was incorporated to avoid measuring engagement in care that could be associated with mpox symptoms or mpox exposure.

Engagement in care was defined as having at least two HIV-associated laboratory tests (CD4 or HIV RNA) or HIV-related care encounters at least 3 months apart during the 12-month window of assessment. We also ran a sensitivity analysis evaluating engagement in care defined as at least one HIV-associated laboratory test (CD4 or HIV RNA) or HIV-related care encounter during the 12-month assessment window. STI screening was defined as having at least one screen for either chlamydia, gonorrhea, or syphilis in the past 12 months. 

### 2.4. Potential Confounders and Other Measures

Additional exposures of interest at the time of mpox diagnosis were CD4 cell count (<200 cells/µL, 200–499 cells/µL, ≥500 cells/µL), virologic suppression (VS; HIV RNA ≤ 200 copies/mL), and history of an STI. The CD4 cell count and HIV RNA laboratory measurements taken closest to the index date during the assessment period were evaluated. History of STI diagnosis was defined as any current or former diagnosis of syphilis, gonorrhea, or chlamydia that occurred after DC Cohort enrollment up until the index date. Antiretroviral therapy (ART) was measured dichotomously as of the index date. Participants with an ART start date in the year prior to the index date or a prior ART start date without an end date were considered to be on ART. 

We explored the relationship between the following additional covariates and mpox diagnosis: year of cohort enrollment, race/ethnicity (non-Hispanic Black, non-Hispanic White, Hispanic/Latino, Other), HIV mode of transmission (sexual vs. non-sexual), time since HIV diagnosis, and HIV care site type (community or hospital). All confounders for cases and controls were measured as of the index date (defined above). Mpox vaccination status was determined by lists acquired from each participant’s HIV care site.

### 2.5. Statistical Analysis

Differences in baseline characteristics between mpox cases and controls were evaluated using chi-square tests for categorical variables and Wilcoxon Rank Sum tests for continuous measures. Categorical variables were presented as frequencies (percentages (%)) and continuous variables as medians (interquartile range (IQR)). Additional measures of engagement in care, such as years since last HIV-associated encounter and concurrent diagnosis within 6 months of mpox, were described in greater detail for identified mpox cases.

We used conditional logistic regression to estimate the risk of mpox based on engagement in care in the past 12 months, history of STI, and STI screening in the past 12 months. Multivariable conditional logistic regression models evaluated the association between (1) prior STI, engagement in care, CD4 (<200 cells/µL), and risk of mpox diagnosis and (2) STI screening, CD4 (<200 cells/µL), engagement in care, and risk of mpox diagnosis. The models were then repeated using CD4 < 500 cells/µL in place of CD4 (<200 cells/µL) to explore the impact of more severe immune dysfunction. All models were adjusted for VS (<200 copies/mL), HIV mode of transmission, race/ethnicity, years since DC Cohort enrollment, and years since HIV diagnosis. 

We excluded healthcare encounters during the 10 days preceding mpox diagnosis as they were more likely to capture care visits related to mpox symptoms. To determine the appropriateness of this 10-day period, we performed a sensitivity analysis in which additional logistic regression analyses within the 12-month window of assessment were shifted to 6 weeks, 2 weeks, and 0 days before mpox diagnosis. 

Statistical Analysis System (SAS) software (version 9.4; SAS Institute, Cary, NC, USA) was used to manage and analyze the data. Statistical significance is considered at *p* < 0.05.

### 2.6. Characterizing Clinical Outcomes of People with Mpox

Our secondary objective was to evaluate demographics, HIV clinical outcomes, and characteristics of participants with mpox. Therefore, we extracted data on demographics, HIV clinical characteristics, and other social determinants of health from the DC Cohort database. To better evaluate cases’ current and ongoing clinical diagnoses and symptoms, ICD10 codes recorded up to 6 weeks before mpox diagnosis were extracted from the EHR and classified into categories based on symptom location, disease severity, and potential to be associated with an STI. Important categories of note are STI-associated diagnoses, general infection symptoms, dermatological condition symptoms (unspecified location), anal disorders, urologic disorders, and other viral infections, and were presented as frequencies (%).

## 3. Results

### 3.1. Demographics: Overall Sample Description

There were 70 cases of mpox among DC Cohort participants in 2022. Among the DC Cohort, there are 7748 male participants, with 5888 having evidence of HIV care between 2015–2023. From the eligible participants, 323 controls were selected, of which 22 served as a control for multiple cases. Of the 70 mpox cases, 3 also served as a control for cases before their mpox diagnosis, leaving 393 unique participants for the analysis. The median age among the study population was 41 years (IQR: 36, 50). Associations between cases and controls were similar at index date [Table 1] in terms of time since HIV diagnosis (Median: 12 years (IQR: 8, 18)) and years enrolled in the DC Cohort (Median: 6 years, (IQR: 2, 8)). The majority of study participants were non-Hispanic Black (72.3%), using private insurance (49.6%), on ART at the index date (86.3%), receiving care at a community-based site (71.5%), and their mode of HIV transmission was documented as sexual (86.0%). Substance use disorders were reported among 35.9%, and alcohol use disorder was found among 17.6% [Table 1]. Cases and controls had similar levels of engagement in care from 2015 to 2022 [Supplemental Table S1]. 

There were significant differences found among cases and controls with regard to low CD4 levels (<200 cells/µL; 7.1% vs. 1.9%; *p* = 0.002) and STI screening (68.6% vs. 55.1%; *p* = 0.04), respectively. There was also a significantly (*p* < 0.0001) higher proportion of cases (71.4%) with a prior STI diagnosis compared to controls (43.3%), and cases were significantly less likely to have an alcohol use disorder (8.6% vs. 20.7%; *p* = 0.01). Cases were more likely to have a viral load >200 copies/mL (*p* = 0.01), although this may be due to a high proportion of controls with missing viral load labs (20.7%). Among the 393 participants, vaccination status was available for 136 (34.6%). Of the participants for whom vaccine information was available, twenty (14.7%) had received at least one dose of the mpox vaccine, of which eleven were mpox cases and nine were controls. However, the exact dates and number of doses could not be confirmed, so whether doses were given before the index date is unknown. 

### 3.2. Conditional Logistic Regression: Risk of Mpox

In unadjusted conditional logistic regression, engagement in care in the past 12 months was not associated with the risk of mpox diagnosis (OR: 1.53 (95% CI: 0.89, 2.64), *p* = 0.12). The same was true after adjusting for the mode of HIV transmission, race/ethnicity, years since DC Cohort enrollment, and years since HIV diagnosis. We used adjusted conditional logistic regression models to further evaluate engagement in care. Models #1 and #2 evaluated low CD4 using a <200 cells/µL cutoff, while models #3 and #4 used a low CD4 cutoff of <500 cells/µL. Given the multicollinearity between prior STI diagnosis and STI screening, STI diagnosis was only included in models #1 and #3, while STI screening was only included in models #2 and #4. All models were adjusted for HIV transmission factor, race/ethnicity, years since DC Cohort enrollment, and years since HIV diagnosis. Engagement in care in the past 12 months was not associated with being diagnosed with mpox in the adjusted models (Model #1 aOR: 1.03 (95% CI: 0.56, 1.91), *p* = 0.93; Model #2 aOR: 1.09 (95% CI: 0.57, 2.06), *p* = 0.80; Model #3 aOR: 1.00 (95% CI: 0.54, 1.83) *p* = 0.99; Model #4 aOR: 1.04 (95% CI: 0.56, 1.96), *p* = 0.90) [Table 2]. These findings remained consistent with the 6-week, 2-week, and 0-day buffer before the mpox diagnosis date [Supplemental Table S2]. 

On the other hand, STI screening in the past 12 months was associated with an increased risk of mpox diagnosis (OR: 2.05 (95% CI: 1.10, 3.82), *p* = 0.02) in the unadjusted model. However, in the multivariable conditional logistic regression model, STI screening was not associated with being diagnosed with mpox after adjusting for the mode of HIV transmission, race/ethnicity, years since DC Cohort enrollment, years since HIV diagnosis, CD4, and engagement in care in the past 12 months (Model #2 aOR: 1.22 (95% CI: 0.56, 2.64), *p* = 0.62; Model #4 aOR: 1.21 (95% CI: 0.56, 2.63), *p* = 0.63). A prior STI was associated with a significantly higher risk of mpox diagnosis in the unadjusted model (OR: 3.98 (95% CI: 1.12, 7.46), *p* < 0.0001) and continued to be associated with increased risk of mpox after adjusting for mode of HIV transmission, race/ethnicity, years since DC Cohort enrollment, years since HIV diagnosis, CD4, and engagement in care in the past 12 months (Model #1 aOR: 3.27 (95% CI: 1.64, 6.49), *p* = 0.001; Model #3 aOR: 3.39 (95% CI: 1.70, 6.75), *p* = 0.001) [Table 2].

Furthermore, low CD4 count was associated with increased odds of mpox diagnosis when both a < 200 cells/µL and a <500 cells/µL cutoff were used (<200 cells/µL OR: 3.93 (95% CI: 1.11, 13.89), *p* = 0.03; <500 cells/µL OR: 1.79 (95% CI: 1.04, 3.09), *p* = 0.04). Similar associations were found in the multivariable logistic regression models that did not account for prior STI screening (Model #2, <200 cells/µL aOR: 4.60 (95% CI: 1.23, 17.11), *p* = 0.02; Model #4, <500 cells/µL aOR: 1.88 (95% CI: 1.05, 3.38), *p* = 0.04). However, low CD4 was not associated with increased odds of mpox diagnosis after adjusting for prior STI diagnosis. (Model #1, <200 cells/µL aOR: 3.35 (95% CI: 0.91, 13.11), *p* = 0.07; Model #3, <500 cells/µL aOR: 1.80 (95% CI: 0.98, 3.31), *p* = 0.06). [Table 2]. 

### 3.3. Clinical Diagnosis of Cases: Diagnosis Code Categories

Descriptions of unique ICD10 diagnoses (n = 208) among cases occurring up to 6 weeks before the mpox diagnosis date were classified based on symptom location, disease severity, and potential to be associated with an STI. The most common diagnoses were STI-associated (14%), such as anogenital herpes, syphilis, chlamydia, and gonorrhea. Chronic conditions comprised 11% of diagnoses. General infection symptoms (including gastrointestinal and constitutional) were the third most common (12%), followed by dermatological issues of an unspecified location, such as rashes or skin lesions (12%) [Supplemental Table S3]. 

## 4. Discussion

In a nested-case control study in a large longitudinal cohort of PWH in Washington, DC, we found that among PWH, engagement in care and STI screening in the past 12 months were not associated with mpox diagnosis. The lack of association between engagement in care and mpox suggests that the high prevalence of HIV among mpox cases nationally may not be due to increased encounters with the healthcare system. In our study, approximately 54.3% of the PWH with mpox were not engaged in HIV-related care in the year before their mpox diagnosis compared to 63.2% of controls; however, the difference was non-significant. This suggests that although patients may not be coming to the clinic regularly, they are still accessing healthcare when receiving an mpox diagnosis. It is possible that the severity of mpox symptoms may lead to healthcare interactions and future analyses should be done to determine whether mild cases of mpox are more likely to go unnoticed. Of note, when we used the alternative, less stringent definition of engagement in care, defined as any HIV encounter or lab in the 12-month assessment period, 82.9% of cases and 68.1% of controls were engaged in care (*p* = 0.01). However, after adjusting for covariates, the associations were non-significant. Given the high prevalence of symptomatic mpox infections, it is possible that people with mpox have a high likelihood of seeking medical care regardless of prior engagement in care. Future studies should examine whether surveillance bias plays a role in asymptomatic infections commonly found among PWH, such as asymptomatic STIs, in which diagnoses can only be made through laboratory testing and may be more easily missed among people not engaged in care. 

Our study found that among PWH, prior STI diagnosis and low CD4 were associated with an increased risk of mpox diagnosis after adjusting for other covariates. Associations between STI diagnosis, CD4 count, and mpox have been found previously [4,5,6,20]. These STI-related ICD codes at the time of mpox diagnosis are not surprising given the association found here between a history of STIs and mpox diagnosis, either due to biological or behavioral connections. However, many of our participants did not have an ICD code for mpox specifically. Therefore, these additional codes at the time of mpox diagnosis may be serving as a proxy for mpox diagnoses during the early days of the outbreak when clinical diagnoses were more uncertain. Future cohort studies should examine these ICD codes as a potential proxy for mpox diagnoses, especially early on in the 2022 outbreak. 

Because it is unlikely that increased encounters with the healthcare system explain the high prevalence of HIV among mpox cases, another clinical or biological association may potentially be contributing. While our findings suggest low CD4 may put PWH at an increased risk for mpox diagnosis, previous studies have concluded low CD4 counts and high HIV RNA viremia are also associated with more severe mpox outcomes [4,5,6,20]. An immunocompromised status may be associated with an increased risk of acquiring mpox with clinical manifestations and an increased risk of severe outcomes, emphasizing the importance of vaccinations among PWH. These findings should be considered when discussing whether mpox should be classified as an opportunistic infection. 

Our study has several limitations. First, we were limited by sample size, with only 70 documented cases of mpox in our cohort. Of note, our cohort does not include a comparison group of individuals without HIV to directly test whether increased contact with the healthcare system among PWH, compared to people without HIV, led to more mpox diagnoses in PWH. Furthermore, all DC Cohort participants are PWH who receive care in Washington, DC. While we had low levels of engagement in care among our participants, our study population is likely known to be more engaged in care than the general population of PWH [19]. Therefore, these findings may not be generalizable to those who do not have an HIV care provider [19]. STI screening and engagement in care were significantly correlated (Pearson Correlation Coefficient: 0.48 (*p* < 0.0001)), which may have introduced error due to multicollinearity in our models. Then, although we found an association between prior STI diagnoses and mpox diagnosis, we were not able to measure the sexual risk behaviors of our participants. History of STI diagnoses is likely serving as a proxy for prior sexual behaviors. Therefore, our findings may suggest an association between high-risk sexual behavior and mpox rather than STIs alone. High-risk sexual behaviors have previously been found to be a risk factor for mpox [6,11,12]. However, this cannot be confirmed within the limitations of our study. Further, we had limited information on the participants’ mpox vaccination status. Therefore, our models could not account for vaccination status, which may have played a role in mpox acquisition among our participants. Vaccination has been found to be the most effective method for controlling mpox outbreaks [21]. While we were not able to incorporate vaccine status, future studies should evaluate the effectiveness of implementing routine mpox vaccination for PWH and inform future prevention guidelines. 

Finally, our study was limited by potential misdiagnoses. This outbreak was the first worldwide outbreak of mpox, and it is possible that cases occurring early in the outbreak were diagnosed as other STI-related or dermatological conditions. The clinical presentation of mpox resembles many common symptoms found in other infections or STIs [15]. Our findings show that many of our mpox cases had received an STI, general infection, or dermatological ICD10 diagnosis in the 6 weeks before being diagnosed with mpox [Supplemental Table S2]. Participants may have also received an mpox diagnosis at another care site and, therefore, would not have shown up as a case in our study. Due to these potential misdiagnoses, it is possible our study did not capture all instances of mpox or captured mpox diagnoses that were not actual cases. As with HIV, the underlying stigma associated with mpox must be addressed. Stereotypical beliefs around populations most impacted by the outbreak, such as MSM, may inhibit engaging in clinical care and lead to further misclassification [22,23]. Messaging around mpox vaccination can go hand in hand with reducing stigma by normalizing preventative measures. These misclassifications of the outcome may have biased our findings towards the null.

## 5. Conclusions

While our study did not find an association between engagement in care and having an mpox diagnosis, we did find that history of an STI diagnosis and lower CD4 counts were both associated with an increased risk of mpox. Although these findings support the conclusion that low CD4 levels are a risk factor for mpox among PWH, immunocompromised status should not be confused with HIV status; therefore, it cannot be concluded that PWH, regardless of their immune status, are at an increased risk for mpox acquisition. These findings support the theory that there is a clinical or biological interface between mpox and HIV and provide evidence that disputes the speculation that the high prevalence of mpox among PWH is due to a surveillance bias. Future studies should further evaluate these biological associations and examine how PWH can be better protected against mpox going forward.

## Figures and Tables

**Table 1:** Description of the Clinical and Demographic Characteristics of Case and Control Study Participants by Mpox Status, DC Cohort (N = 393).

	Overall	Cases	Controls	*p*-Value
	(N = 393)	(n = 70)	(n = 323)	
	N (%)	N (%)	N (%)	
Age [Years], Median (IQR)	41 (36, 50)	41 (36, 48)	41 (36, 50)	0.69
Time Since HIV Diagnosis [Years], Median (IQR)	12 (8, 18)	13 (10, 17)	12 (8, 18)	0.41
Time Since DC Cohort Enrollment [Years], Median (IQR)	6 (2, 8)	6 (4, 8)	5 (2, 8)	0.23
Gender Identity				
Male	393 (100.0)	70 (100.0)	323 (100.0)	1.00
Ethnicity and race				0.41
Non-Hispanic White	48 (12.2)	6 (8.6)	42 (13.0)	
Non-Hispanic Black	284 (72.3)	49 (70.0)	235 (72.8)	
Hispanic/Latino	40 (10.2)	10 (14.3)	30 (9.3)	
Other/Unknown	21 (5.3)	7 (7.1)	16 (5.0)	
Insurance Status				0.33
Public ^A^	134 (34.1)	29 (41.4)	105 (32.5)	
Private	195 (49.6)	32 (45.7)	163 (50.5)	
Other/Unknown	64 (16.3)	9 (12.9)	55 (17.0)	
Care Site Type				0.99
Hospital	112 (28.5)	20 (28.6)	92 (28.5)	
Community	281 (71.5)	50 (71.4)	231 (71.5)	
Mode of HIV Transmission				0.15
Sexual	338 (86.0)	64 (91.4)	274 (84.8)	
Non-Sexual ^B^	55 (14.0)	6 (8.6)	49 (15.2)	
Alcohol Use Disorder ^C^	69 (17.6)	6 (8.6)	63 (19.5)	**0.03**
Substance Use Disorder ^D^	141 (35.9)	25 (35.7)	116 (35.9)	0.97
Antiretroviral Therapy	339 (86.3)	63 (90.0)	276 (85.5)	0.32
HIV RNA [copies/mL], Median (IQR) E	20 (20, 70)	20 (20, 420)	20 (20, 50)	
HIV RNA Categories				0.05
≤200 copies/mL	376 (70.2)	50 (71.4)	226 (70.0)	**0.01**
>200 copies/mL	44 (11.2)	14 (20.0)	30 (9.3)	
Unknown	73 (18.6)	6 (8.6)	67 (20.7)	
CD4 [cells/µL], Median (IQR) E	648 (485, 840)	572 (413, 738)	665 (501, 869)	
CD4 Categories				**0.01**
<200 cells/µL	11 (2.8)	5 (7.1)	6 (1.9)	**0.002**
200–499 cells/µL	78 (19.9)	20 (28.6)	58 (18.0)	
≥500 cells/µL	235 (59.8)	40 (57.1)	195 (60.4)	
Unknown	69 (17.6)	5 (7.1)	64 (19.8)	
Mpox Vaccination Status At Least One Vaccine Dose No Vaccine Dose Unknown	20 (5.1) 116 (29.5) 257 (65.4)	5 (7.1) 54 (77.1) 11 (15.7)	9 (2.8) 62 (19.2) 252 (78.0)	**<0.0001**
History of STI Diagnosis	190 (48.4)	50 (71.4)	140 (43.3)	**<0.0001**
STI Screening ^F,G^	226 (57.5)	48 (68.6)	178 (55.1)	**0.04**
Engagement in HIV-Associated Care ^F^	151 (38.4)	32 (45.7)	119 (36.8)	0.17

^A^ DC Alliance, Medicaid, Medicare, Ryan White/ADAP, Other public funding; ^B^ IDU, Perinatal, Other, Missing; ^C^ Former or current alcohol abuse; ^D^ Any former or current substance use excluding smoking and alcohol use; ^E^ Based on the most recent lab measurement during the 12-month window of assessment; ^F^ In the 12 months prior to 10 days before index date; ^G^ Screening for at least one of the following: gonorrhea, chlamydia, syphilis.

**Table 2:** Conditional Logistic Regression Models of Mpox Diagnosis (N = 398).

	Unadjusted Conditional OR (95% CI)	Model #1 Conditional aOR (95% CI) ^A^	Model #2 Conditional aOR (95% CI) ^B^	Model #3 Conditional aOR (95% CI) ^C^	Model #4 Conditional aOR (95% CI) ^D^
Antiretroviral Therapy	2.1 (0.78, 5.51)	N/A	N/A	N/A	N/A
HIV Viral Suppression ≤200 copies/mL >200 copies/mL Unknown	REF 1.89 (0.95, 3.76) 0.34 (0.13, 0.88)	N/A	N/A	N/A	N/A
CD4 Count				N/A	N/A
<200 cells/µL	3.93 (1.11, 13.89)	3.35 (0.91, 13.11)	4.60 (1.23, 17.11)		
≥200 cells/µL Unknown	REF 0.28 (0.10, 0.77)	REF 0.39 (0.13, 1.15)	REF 0.35 (0.11, 1.12)		
CD4 Count <500 cells/µL ≥500 cells/µL Unknown	1.79 (1.04, 3.09) REF 0.32 (0.11, 0.88)	N/A	N/A	1.80 (0.98, 3.31) REF 0.43 (0.15, 1.27)	1.88 (1.05, 3.38) REF 0.39 (0.12, 1.24)
Prior STI Diagnosis	3.98 (1.12, 7.46)	3.27 (1.64, 6.49)	N/A	3.39 (1.70, 6.75)	N/A
STI Screening ^E^	2.05 (1.10, 3.82)	N/A	1.22 (0.56, 2.64)	N/A	1.21 (0.56, 2.63)
Engagement in Care ^E^	1.53 (0.89, 2.64)	1.03 (0.56, 1.91)	1.09 (0.57, 2.06)	1.00 (0.54, 1.83)	1.04 (0.56, 1.96)

^A^ Model #1 is adjusted for engagement in care in the past 12 months, prior STI diagnosis, CD4 ≥ 200 copies/mL, mode of HIV transmission, race/ethnicity, years since DC Cohort enrollment, and years since HIV diagnosis; ^B^ Model #2 is adjusted for engagement in care in the past 12 months, STI screening in the past 12 months, CD4 ≥ 200 copies/mL, mode of HIV transmission, race/ethnicity, years since DC Cohort enrollment, and years since HIV diagnosis; ^C^ Model #3 is adjusted for engagement in care in the past 12 months, prior STI diagnosis, CD4 ≥ 500 copies/mL, mode of HIV transmission, race/ethnicity, years since DC Cohort enrollment, and years since HIV diagnosis; ^D^ Model #4 is adjusted for engagement in care in the past 12 months, STI screening in the past 12 months, CD4 ≥ 500 copies/mL, mode of HIV transmission, race/ethnicity, years since DC Cohort enrollment, and years since HIV diagnosis; ^E^ In the 12 months to 10 days before the index date.

## Data Availability

Per DC Cohort protocols, data are available upon request and approval of the DC Cohort Executive Committee. Interested parties should email the Principal Investigator at acastel@gwu.eu.

## Supplementary Materials

The following supporting information can be downloaded at: https://www.mdpi.com/article/10.3390/pathogens13020117/s1, Table S1: Prior Engagement in Care; Table S2: Sensitivity Analysis of Index Date Buffer; Table S3: Associated ICD codes at time of mpox diagnosis.
