# Supplementary material for: Characterizing Indicators of Engagement in HIV-Associated Healthcare and Clinical Outcomes among People with HIV and Mpox in Washington, DC: A Nested Case-Control Study of the DC Cohort
Source: Pathogens. 2024 Jan 27;13(2):117. doi: 10.3390/pathogens13020117 (PMC10892472; doi:10.3390/pathogens13020117)
Supplement: Supplementary file 1 [file pathogens-13-00117-s001.zip › pathogens-2814498-supplementary.pdf]

SUPPLEMENTAL MATERIAL

Supplemental Table S1. Overview of participants engaged in care in the years leading up to mpox diagnosis.

	Proportion Engaged in Care in the Year Prior (N (%))							
	2015	*2016*	*2017*	*2018*	*2019*	*2020*	*2021*	*2022*
**Cases**	17 (24.3)	22 (31.4)	39 (55.7)	43 (61.4)	44 (62.9)	48 (68.6)	41 (58.6)	52 (74.3)
**Controls**	80 (24.8)	89 (27.6)	133 (41.2)	167 (51.7)	191 (59.1)	201 (62.2)	196 (60.7)	219 (67.8)

Supplemental Table S2. Association between participant factors and having an mpox diagnosis using different buffers before the index date.

	Index Date			
	6 Weeks Prior to Index Date	2 Weeks Prior to Index Date	10 Days Prior to Index Date	Index Date (0-day buffer)
	OR (95% CI)	OR (95% CI)	OR (95% CI)	OR (95% CI)
Model #1: Engagement in Care[A,E]	0.85 (0.45, 1.59)	1.01 (0.54, 1.88)	1.03 (0.56, 1.91)	1.73 (0.91, 3.28)
Model #2: Engagement in Care[B,E]	0.89 (0.47, 1.71)	1.09 (0.57, 2.07)	1.09 (0.57, 2.06)	1.57 (0.81, 3.04)
Model #3: Engagement in Care[C,E]	0.82 (0.44, 1.51)	0.97 (0.53, 1.79)	1.00 (0.54, 1.83)	1.66 (0.89, 3.10)
Model #4: Engagement in Care[D,E]	0.85 (0.45, 1.60)	1.04 (0.55, 1.96)	1.04 (0.56, 1.96)	1.49 (0.78, 2.83)

[A]Model #1 is adjusted for Engagement in care in the past 12 months, prior STI diagnosis, CD4 ≥ 200 copies/mL, mode of HIV transmission, race/ethnicity, years since DC Cohort enrollment, years since HIV diagnosis; [B]Model #2 is adjusted for Engagement in care in the past 12 months, STI screening in the past 12 months, CD4 ≥ 200 copies/mL, mode of HIV transmission, race/ethnicity, years since DC Cohort enrollment, years since HIV diagnosis; [C]Model #3 is adjusted for Engagement in care in the past 12 months, prior STI diagnosis, CD4 ≥ 500 copies/mL, mode of HIV transmission, race/ethnicity, years since DC Cohort enrollment, years since HIV diagnosis; [D]Model #4 is adjusted for Engagement in care in the past 12 months, STI screening in the past 12 months, CD4 ≥ 500 copies/mL, mode of HIV transmission, race/ethnicity, years since DC Cohort enrollment, years since HIV diagnosis; [E]In the 12 months to 10 days before the index date

Supplemental Table S3. Additional ICD codes recorded for cases up to 6 weeks prior to the mpox diagnosis date.

**Category of Diagnosis**	**Overall (N (%))**
**STI-Associated Diagnoses**	33 (13.4)
**Other Viral**	22 (8.9)
**Anal Disorders**	23 (9.3)
**Urologic Disorders**	13 (5.3)
**Dermatological Issue**	28 (11.3)
**General Infection Symptoms**	32 (13.0)
**Muscle Pain/Injury/Dysfunction**	17 (6.9)
**Screening**	6 (2.4)
**Encounter for Immunization**	15 (6.1)
**Endocrine & Metabolic**	2 (0.8)
**Hematologic**	2 (0.8)
**Substance Use**	5 (2.0)
**Mood Disorder**	8 (3.2)
**Chronic Conditions**	30 (12.1)
**Other**	11 (4.5)
**ICD 10 Codes**	**Category Specific (N (%))**
**STI-Associated Diagnoses**	
Syphilis	7 (21.2)
A51.49, A51.5, A53.0,A53.9	
Gonococcal Infection	2 (6.1)
A54.9	
Chlamydial Infection	2 (6.1)
A56.3, A74.9	
Anogenital Herpes	8 (24.2)
A60.00, A60.1, A63.0, B00.2, B00.9	
Scabies	1 (3.0)
B86	
Suspected STI Exposure	13 (39.4)
Z11.3; Z20.2; R36.9	
**Other Viral**	
COVID-19	2 (9.1)
U07.1	
COVID-19 Exposure	8 (36.4)
Z20.822	
Exposure to Communicable Disease	1 (4.5)
Z20.89	
Hepatitis B or C	4 (18.2)
B17.10, B18.1, B18.2, B19.20	
Influenza	1 (4.5)
J11.1	
Unspecified Viral Infection	3 (13.6)
B09, B34.9	
Upper Respiratory/Otic	3 (13.6)
H60.92, J02.9	
**Anal Disorders**	
Anorectal Lesion	1 (4.3)

K62.6	
Colon Neoplasm, Benign	1 (4.3)
D12.6	
Constipation	4 (17.4)
K59.00	
Hemorrhoids	2 (8.7)
K64.8	
Rectal Pain	11 (47.8)
K62.89	
Rectal Discharge	2 (8.7)
R19.8	
Unspecified Anorectal Disease	2 (8.7)
K62.9, R85.619	
**Urologic Disorders**	
**Dysuria/Hematuria**	3 (23.1)
R30.0, R31.9	
**Erectile Dysfunction**	1 (7.7)
N52.9	
**Genital Lesion**	4 (30.8)
N48.5, N48.89	
**Proctitis/Urethritis**	2 (15.4)
K51.218, N34.2	
**Unspecified**	3 (23.1)
N50.89, N50.9, N47.2	
**Dermatological Issue**	
**Rash**	16 (57.1)
R21	
**Skin Infection**	10 (35.7)
L08.9, L98.9, L03.211, B36.9	
**Other**	2 (7.1)
L21.9	
**General Infection Symptoms**	
**Constitutional Symptoms**	27 (84.4)
R69, R68.89, R68.83, R59.0, R53.83, R51.9, R50.9, G43.909	
**Gastrointestinal Symptoms**	3 (9.4)
R11.0, R19.7, K92.1	
**Respiratory**	2 (6.3)
J20.9, J30.9	
**Encounter for Immunization**	
Z23, Z71.85, Z71.89	
**Encounter for Screening**	
Z00.00, Z12.11, Z12.5, Z13.6, Z13.9	
**Mood Disorder**	
**Adjustment Disorder**	2 (25.0)
F43.20, F43.23	
**Anxiety**	2 (25.0)
F41.1, F41.9	
**Depression**	3 (37.5)
F32.9, F33.40	
**Insomnia**	1 (12.5)
F51.05	
**Muscle Pain/Injury/Dysfunction**	
**Abdominal Pain**	3 (17.6)

R10.31, R10.9	
**Chest Pain**	3 (17.6)
R07.89, R07.9	
**Musculoskeletal Injury or Pain**	11 (64.7)
G72.9, G89.29, M54.2, M54.50, M54.9, M62.82, M79.89, M84.452A, S16.1XXA, S29.9XXA, S39.012A	
**Chronic Conditions**	
E11.9, E66.9, E78.2, E78.5, G62.9, I10, I25.10, I50.9, I70.0, J45.20, K21.9, K76.0, N13.5, N18.31, N18.4, N18.9, N28.1, R00.0, R03.0, T86.19	
**Substance use**	
F10.20, F10.99, F14.10, F15.10	
**Hematologic**	
D50.9, D64.9	
**Endocrine & Metabolic**	
E53.8, E87.1	
**Other**	
E86.0, F17.200, H26.9, H52.4, J35.1, K11.6, K14.6, K14.8, R06.02, R63.5, T46.4X5S	
